# Systematic Review and Meta-analysis on the Effect of Soy on Thyroid Function

**DOI:** 10.1038/s41598-019-40647-x

**Published:** 2019-03-08

**Authors:** Jemiliat Otun, Amirhossein Sahebkar, Linda Östlundh, Stephen L. Atkin, Thozhukat Sathyapalan

**Affiliations:** 1 Academic Diabetes, Endocrinology and Metabolism, Hull York Medical School, University of Hull, Hull, UK; 2 Neurogenic Inflammation Research Center, Mashhad University of Medical Sciences, Mashhad, Iran; 3 Biotechnology Research Center, Pharmaceutical Technology Institute, Mashhad University of Medical Sciences, Mashhad, Iran; 4 School of Pharmacy, Mashhad University of Medical Sciences, Mashhad, Iran; 5 National Medical Library, College of Medicine and Health Sciences, UAE University, Al Ain, UAE; 6 Weill Cornell Medicine, Doha, Qatar

## Abstract

Soy foods have had an important dietary role in Asian countries for centuries, and in recent years they have become increasingly popular in Western countries as a result of their suggested health benefits. Nevertheless, there are some concerns that soy can have a negative effect on thyroid function and can alter the levels of thyroid hormones. The aim of this systematic review was to investigate the link between soy or soy product consumption and thyroid function via the measurement of thyroid hormone levels. A systematic review and meta-analysis was undertaken on all randomised controlled trials of studies including soy as an intervention and where free triiodothyronine (fT3), free thyroxine (fT4) and thyroid stimulating hormone (TSH) was measured. The search included PubMed, MEDLINE, EMBASE, Cochrane and sources for the grey literature. Quantitative data synthesis was performed using a random-effects model, with standardized mean difference (SMD) and 95% confidence interval as summary statistics. A total of 18 articles were suitable for review. The meta-analysis showed no significant changes in fT3 (WMD: 0.027 pmol/L, 95% CI: −0.052, 0.107, *p* = 0.499; I^2^: 55.58%), fT4 (WMD: −0.003 pmol/L, 95% CI: −0.018, 0.011, *p* = 0.656; I^2^: 87.58%) while an elevation in TSH levels was observed (WMD: 0.248 mIU/L, 95% CI: 0.001, 0.494, *p* = 0.049; I^2^: 80.31%) levels with soy supplementation. There was no evidence of publication bias. Soy supplementation has no effect on the thyroid hormones and only very modestly raises TSH levels, the clinical significance, if any, of the rise in TSH is unclear.

## Introduction

Soy has been a staple in the diet in South and East Asian countries for many years and has become popular in Western countries as a result of the suggested health benefits that include protection against osteoporosis^1^, cardiovascular diseases^2^, diabetes^3,4^, breast cancer^5^ and prostate cancer^6^.

The two main components of soy thought to be responsible for the proposed health benefits are soy protein and the soy isoflavones. It is believed that isoflavones present in soy are the main active compounds that produce both hormonal and non-hormonal effects^7^. Isoflavones are a subclass of common plant compounds called flavonoids. They usually exist in food as biological inactive forms, such as genistin and daizin, which are present in soy as β-D-glycosides. These glycosides can be hydrolyzed and deconjugated by β-glycosidases synthesized by intestinal bacteria to form biologically active aglycone forms of isoflavones, such as genistein and daidzein. Public interest in the health benefits of soy isoflavones has led to the development of isoflavone supplements and the fortification of foods with isoflavones^7,8^.

However, there is speculation that for certain subsets of the population soy foods and isoflavones may be detrimental. Concern has been expressed that soy may adversely affect thyroid function in susceptible individuals by interfering with the absorption of synthetic thyroid hormone^9,10^. Although the anti-thyroid effect of soy has been investigated in animals for decades^11^, the identification of several cases of thyroid enlargement in infants consuming soy formula in the early 1960s, over 60 years ago, became apparent^12^. It has been shown that isoflavones inhibit the activity of thyroid peroxidase (TPO), an enzyme involved in the synthesis of triiodothyronine (T3) and thyroxine (T4), *in vivo* in rats^13^ and *in vitro*^14^.

There is some evidence to suggest that soy consumption is associated with thyroid disorders such as hypothyroidism, goitre, and autoimmune thyroid disease^9^. The potential public health implications of the possible anti-thyroid effects of soy is important since as many as 10% of postmenopausal women, for whom soy foods have particular appeal, may be hypothyroid, and a large proportion of these may be undiagnosed^15^. Thus, it is crucial to clarify the relationship between soy and thyroid function. There was a review of literature on the effect of soy protein and soybean isoflavones on thyroid function in healthy adults and hypothyroid patients in 2006, although this was not a prospectively registered systematic review^16^. In 2015, the European Food Safety Authority (EFSA) conducted a risk assessment that included the effect of soy protein and soy isoflavones on thyroid function^17^, however, there have been a number of randomised controlled studies published after this review^18–20^. The EFSA concluded soy isoflavones were without effect on thyroid function.

## Material and Methods

### Search Strategy

A comprehensive, systematic search for information was conducted in several academic databases as well as in sources of grey literature based on following the main search string:

((TSH OR thyrotropin OR triiodothyronine OR thyroxine OR “Iodide peroxidase” OR “TPO protein” OR “TPO proteins” OR thyroid* OR T4 OR T3) OR hypothyroid* OR levothyroxine) AND (equol OR phytoestrogen*OR genestein OR genistein OR daidzein OR glycitein OR soy* OR genistin OR “lipofundin s” OR “sodium-iodide symporter” OR GCP OR isoflavones).

Pre-searches to identify relevant search terms, search strategies and information sources were conducted in May–June 2016, and the main search was carried out in July–August 2016. Additional searches for grey literature was later conducted during August- September 2016.

PubMed was used to systematically develop and test the search string, which later was adapted and applied to MEDLINE (WOS), EMBASE (OVID), and Cochrane.

All terms were searched in the fields “Abstract” and “Article Title” and in MeSH/Subject Headings/Thesaurus when available. No filters or limitations were applied in order to retrieve the best possible result and to avoid excluding pre- indexed materials.

All references were uploaded to the reference management software EndNote and transferred to the systematic review software Covidence for de-duplication, screening and data extraction.

In addition to the search in academic databases, ProQuest Dissertation and Thesis, Ethos, Open Grey, The New York Academy of Grey Literature Reports and Clinical Trials were searched for grey materials. The sources were selected based on pre-searches and includes American, European as well as global publications. Due to the lack of advanced search features in many of the grey resources, broader search strings than the one used in the academic databases had to be applied.

An updated search in PubMed with a publication filter for the period August 2016–August 2018 was additionally conducted in August 2018 to include the most recent papers published.

### Study Selection

Original studies were included if they met the following inclusion criteria: (i) Adult trials, (ii) being a randomized controlled trial with either parallel or cross-over design, (iii) investigating the impact of soy products e.g. isoflavones, genistein and/or daizein on plasma/serum concentrations of TSH, fT3 and fT4, and, (iv) presentation of sufficient information on TSH, fT3 and fT4 concentrations at baseline and at the end of follow-up in each group or providing the net change values. Exclusion criteria were: (i) Animal trials, (ii) Child/Adolescent Trials (iii) observational studies with case-control, cross-sectional or cohort design, and (iv) lack of sufficient information on baseline or follow-up TSH, fT3 and fT4 concentrations.

### Data extraction

Eligible studies were reviewed and the following data were abstracted by two co-authors. Any disagreements were resolved through discussion and consultation with a third co-author: (1) first author’s name; (2) year of publication; (3) country were the study was performed; (4) study design; (5) number of participants in the intervention and control/placebo groups; (6) intervention assigned to the control group if any; (7) soy product type (isoflavones, genistein and/or daidzein) and dose of soy supplement; (8) treatment duration; (9) age, gender and body mass index (BMI) of study participants; and (9) data regarding baseline and follow-up plasma concentrations of TSH, fT3 and fT4.

### Quantitative Data Synthesis

Meta-analysis was conducted using Comprehensive Meta-Analysis (CMA) V2 software (Borenstein M *et al*. Comprehensive Meta-analysis Version 2, Biostat, Englewood NJ, 2005). A random-effects model (using DerSimonian-Laird method) and the generic inverse variance weighting method were used to compensate for the heterogeneity of studies in terms of study design, treatment duration, and the characteristics of populations being studied^21^. Standard deviations (SDs) of the mean difference were calculated using the following formula: SD = square root [(SD_pre-treatment_)^2^ + (SD_post-treatment_)^2^ − (2 R × SD_pre-treatment_ × SD_post-treatment_)], assuming a correlation coefficient (R) = 0.5. Where standard error of the mean (SEM) was only reported, standard deviation (SD) was estimated using the following formula: SD = SEM × sqrt (*n*), where *n* is the number of subjects. All fT3, fT4 and TSH values were collated in pmol/L, pmol/L and mIU/L, respectively. Effect sizes were expressed as weighted mean difference (WMD) and 95% confidence interval (CI). In case of studies with multiple treatment arms and a single control group, the number of subjects in the control group was divided into equal parts to avoid double counting of a single individual in the meta-analysis. In order to evaluate the influence of each study on the overall effect size, a sensitivity analysis was conducted using the leave-one-out method (i.e., removing one study each time and repeating the analysis. Since pre-test-post-test correlation coefficients (R) were not reported by the studies, an (R)value of 0.5 was assumed through this meta-analysis, as this value is a conservative estimate for R which ranges between 0 and 1. In order to check if the R value can alter the results of meta-analysis, sensitivity analyses were performed by repeating the analysis with R values of 0.2, 0.3, 0.7 and 0.8. The results showed the robustness of the pooled estimate with different R values.

### Publication bias

Evaluation of funnel plot, Begg’s rank correlation and Egger’s weighted regression tests were employed to assess the presence of publication bias in the meta-analysis. When there was evidence of funnel plot asymmetry, potentially missing studies were imputed using the “trim and fill” method^22^. This method imputes the potentially missing studies on either side of the funnel plot, and then re-computes the effect size considering inclusion of potentially missing studies^22^.

## Results

4925 references were found through the literature search, with 1816 references remaining after de-duplication. Additionally, 145 papers were identified in the updated PubMed search and hand screened. A total of 4 new papers were selected to be included in the study. A full search log, including search string, results and notes can be found in Appendix 1.

**Figure 1:**
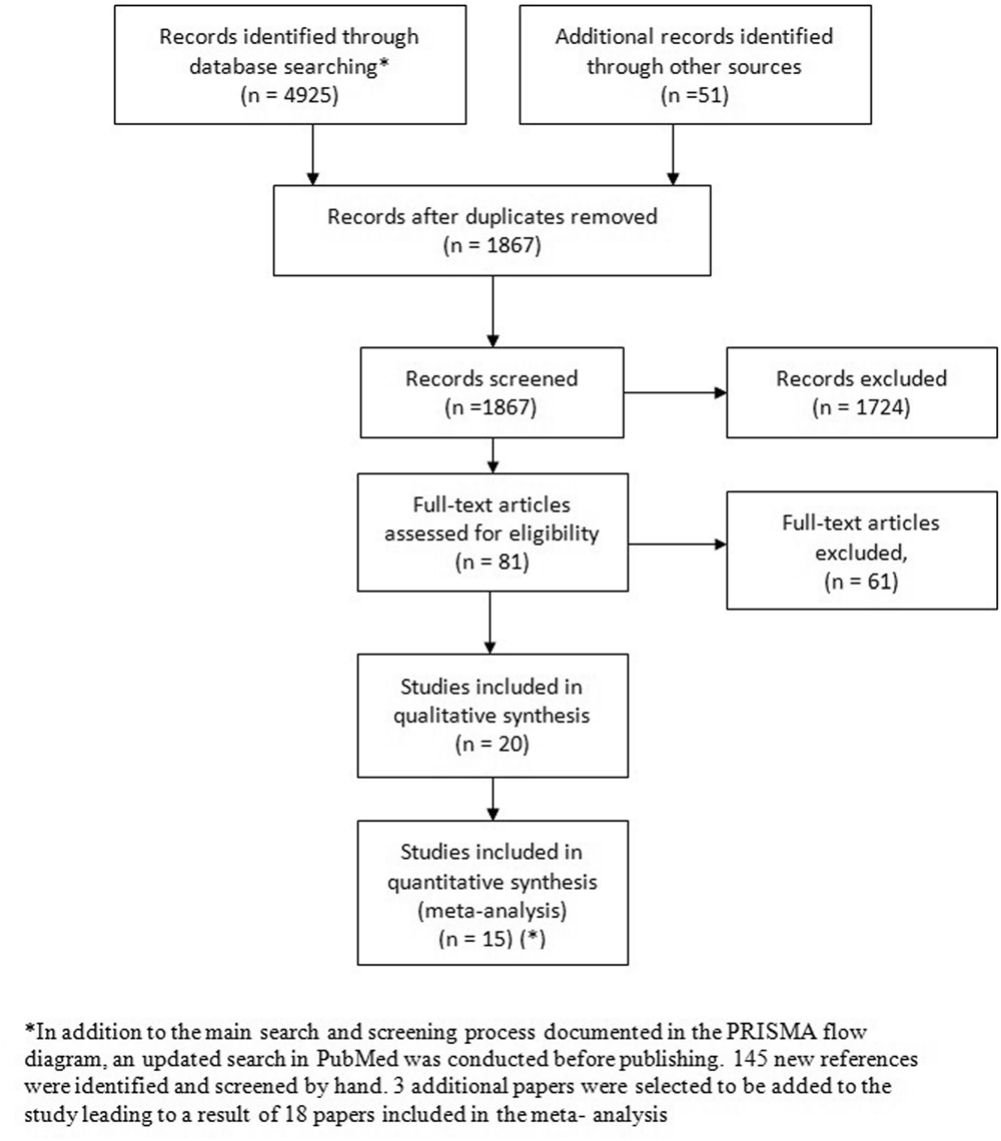
PRISMA Studies Selection Flow Diagram. *In addition to the main search and screening process documented in the PRISMA flow diagram, an updated search in PubMed was conducted before publishing. 145 new references were identified and screened by hand. 3 additional papers were selected to be added to the study leading to a result of 18 papers included in the meta- analysis.

In total, after the initial and updated searches, 84 references were identified in the TI/AB screening, 23 were remaining after full text screening and a final of 18 selected to be included in the meta-analysis. A PRISMA flow diagram of the screening and selection process can be found in Fig. 1.

The interventions used in the studies were mainly food supplements containing soy isoflavones, soy extract, soy protein, daidzein-rich isoflavones and genistein alone. The doses used in the studies ranged from 40–200 mg/day. In comparison, in one survey based study older Japanese adults consumes approximately 25–50 mg of isoflavones (expressed as aglycone equivalents) per day^23^. We included only fT3 and fT4 since they are the metabolically active form of thyroid hormones since they are not bound to plasma proteins including thyroid binding globulin.

Since the primary endpoints in most studies included were not related to thyroid function except seven studies^19,20,24–28^, the sample sizes were mainly determined on the basis of expected changes in other endpoints. Data from 18 studies were included in the meta-analysis^3,18–20,24–37^. Out of this, two studies included both men and women^19,20^, 3 studies included men only^25,29,36^, whilst the other studies included only women. Two studies looked into changes in thyroid function in patients with subclinical hypothyroidism^19,20^.

### Quantitative data synthesis

**Figure 2:**
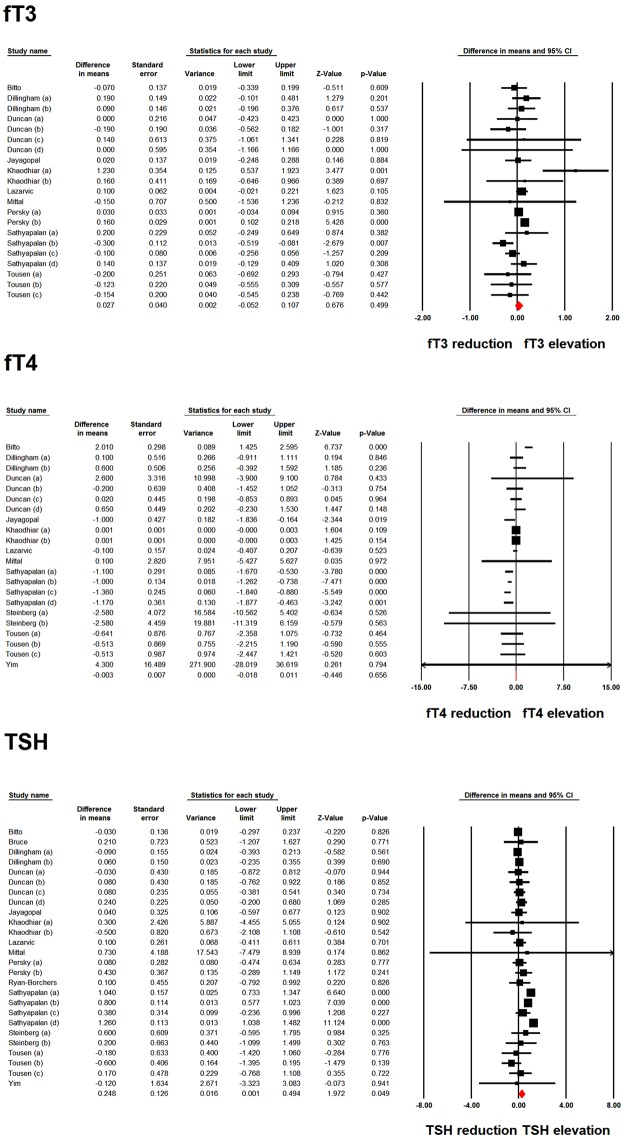
Forest plot displaying weighted mean difference and 95% confidence intervals for the impact of soy products on fT3, fT4 and TSH levels.

**Figure 3:**
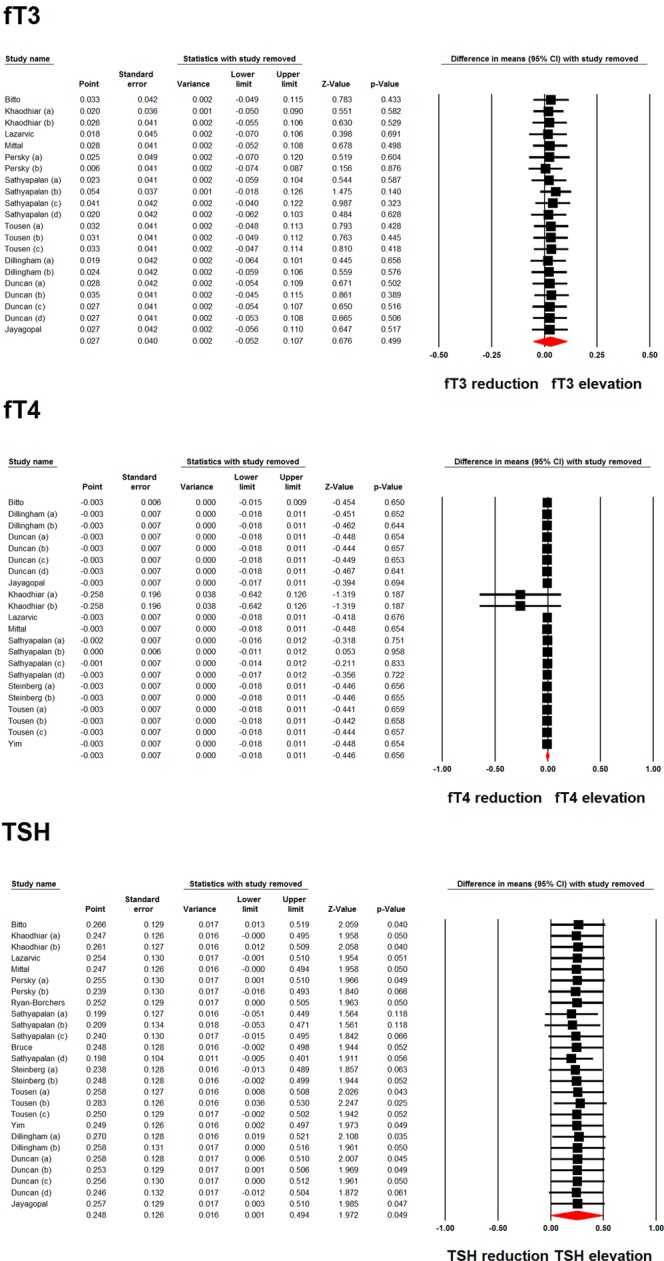
Leave-one-out sensitivity analysis of the impact of soy products on fT3, fT4 and TSH levels.

Meta-analysis of data from 21 and 22 treatment arms did not reveal any significant effect of soy product consumption on fT3 (WMD: 0.027 pmol/L, 95% CI: −0.052, 0.107, *p* = 0.499; I^2^: 55.58%) and fT4 (WMD: −0.003 pmol/L, 95% CI: −0.018, 0.011, *p* = 0.656; I^2^: 87.58%). However, analysis of 26 treatment arms revealed a significant elevation in TSH levels following soy product consumption (WMD: 0.248 mIU/L, 95% CI: 0.001, 0.494, *p* = 0.049; I^2^: 80.31%) (Fig. 2). Iterative removal of included study arms from the meta-analysis did not reveal sensitivity of the pooled effect size estimate to any single treatment arm in the meta-analyses of fT3 and fT4. However, the meta-analysis of TSH was sensitive to one of the included treatment arms (Fig. 3).

We have performed subgroup analysis of TSH for potential confounding factors and sources of heterogeneity between studies which included gender, menopause status, health status at baseline, treatment duration and presence of subclinical hypothyroidism at baseline (Table 1).

**Table 1:** Subgroup analysis for changes in TSH.

		WMD (95% CI)	*p*-Value	*Between group p-value*
Gender	Male	0.004 (−0.191, 0.199)	0.968	0.004
	Female	0.232 (−0.087, 0.552)	0.154	
Menopausal status	Premenopausal	0.020 (−0.565, 0.606)	0.946	0.580
	Postmenopausal	0.184 (−0.098, 0.466)	0.201	
Health status at baseline	Healthy	0.131 (−0.108, 0.371)	0.282	0.121
	Not healthy	0.493 (0.104, 0.883)	0.013	
Treatment duration	<3 months	0.006 (−0.180, 0.191)	0.952	0.710
	>3 months	0.319 (0.034, 0.604)	0.028	
Subclinical hypothyroidism at baseline	Yes	0.692 (0.333, 1.052)	<0.001	0.032
	No	0.199 (−0.074, 0.472)	0.154	

Subgroup analysis of changes in TSH reveals treatment duration of more than or equal to three months (WMD (95% CI) 0.319 (0.034, 0.604; p value 0.028) and presence of subclinical hypothyroidism at baseline (WMD (95% CI) 0.692 (0.333, 1.052; p value < 0.001) were significant.

### Publication bias

**Figure 4:**
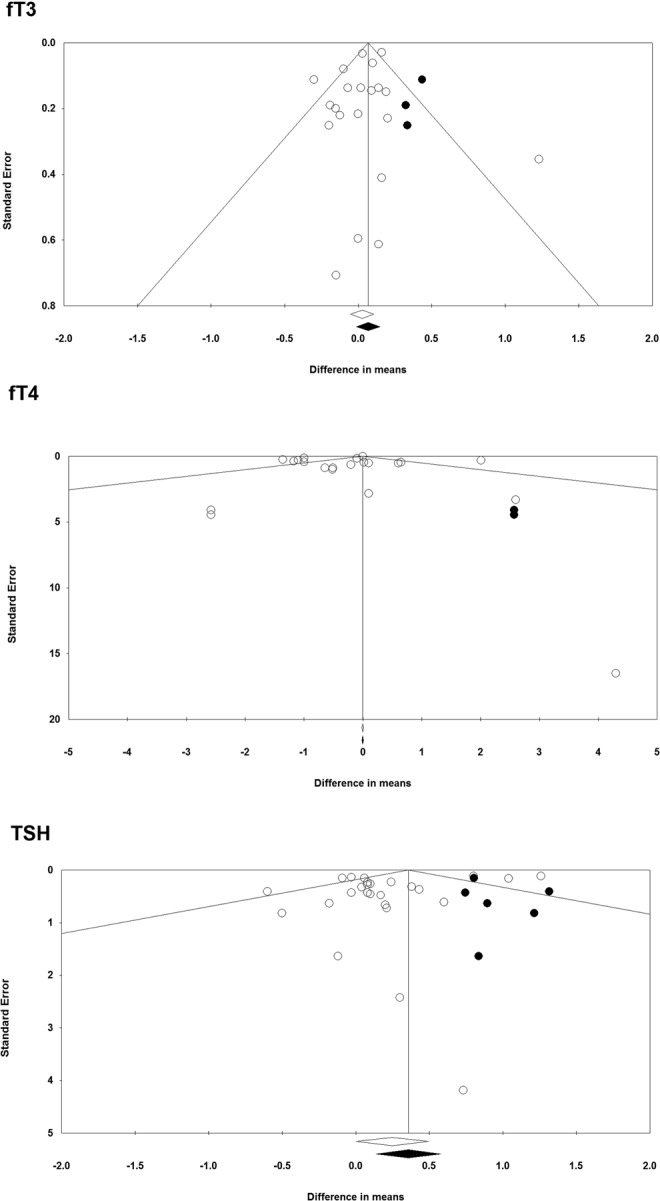
Funnel plot detailing publication bias in the studies reporting the impact of soy products on fT3, fT4 and TSH levels.

Visual inspection of Begg’s funnel plots revealed asymmetry in the meta-analyses of soy product’s effects on fT3, fT4 and TSH levels. Using trim and fill correction, 3, 2 and 6 potentially missing studies were imputed (Fig. 4). Imputed effect sizes and the results of Begg’s rank correlation and Egger’s regression tests are summarized in Table 2.

**Table 2:** Imputed effect sizes and the results of Begg’s rank correlation and Egger’s regression tests for the meta-analysis of Soy’s effects on thyroid hormones.

	n^a^	WMD	95% CI	*P-value* ^*b*^	*P-value* ^*c*^
fT3	3	0.068	−0.012, 0.148	0.381	0.374
fT4	2	−0.003	−0.018, 0.011	0.338	0.224
TSH	6	0.358	0.139, 0.577	0.067	0.065

^a^The number of imputed studies according to the trim and fill correction method; ^*b*^Begg’s rank correlation test; ^*c*^Egger’s weighted regression test.

## Discussion

This systematic review showed that there was a significant change in TSH as a result of soy protein and/or isoflavones supplementation though with no significant changes in fT3 and fT4 levels suggesting that whilst soy can adversely affect thyroid function, clinically this may not be significant. This is the converse reported from the EFSA risk assessment on 2015 that did not show that food supplements containing isolated isoflavones caused any significant effects on thyroid function in peri- or post-menopausal women^17^; however, new randomised controlled trials have been published since this report.

A study that examined the effect of soy isoflavones in patients with type 2 diabetes and hypogonadism showed a significant increase in TSH and reduction in fT4 with 12 weeks of combined soy protein and isoflavone supplementation, though none developed subclinical or overt hypothyroidisim^36^. Another study published recently comparing soy protein and casein protein without soy isoflavones in patients with subclinical hypothyroidism reported no significant changes in thyroid function^19^.

In one of the studies in patients with subclinical hypothyroidism comparing the effect of low dose phytoestrogen (2 mg comparable to Western diet) with higher dose phytoesterogen (comparable to a vegetarian diet), there were no significant changes in thyroid function test as a group. However, of the 60 patients, six of the 52 female subjects (11.5%) progressed into overt hypothyroidism after the 16-mg isoflavone arm of the 6-month period showing a 3-fold increase (standardized rate ratio of 3.6 (95% confidence interval = 1.9, 6.2)) in progression from subclinical to overt hypothyroidism^20^.

Soy containing food products have been potentially implicated in deterioration of thyroid function. Goitre has been seen in infants fed soy formula; this is usually reversed by changing to cows’ milk or iodine-supplemented diets^12,38^. *In vitro* research^14,39^ and studies in rodents^13,40^ supplemented with isolated isoflavones suggests that soy can have an anti-thyroid effect. There are concerns that they may compromise thyroid function^14,41^, an issue for patients with subclinical or overt hypothyroidism, particularly during pregnancy^42–44^.

*In vitro* studies have demonstrated that isoflavones inhibit thyroid peroxidase (TPO), an enzyme involved in the synthesis of T_3_ and T_4_^14^.

Subgroup analysis of changes in TSH reveals treatment duration of more than or equal to three months and presence of subclinical hypothyroidism at baseline were significant. This suggests that soy could have more effect on people with compromised thyroid function at baseline or having various disease states or taken for a longer duration.

Limitations of the present meta-analysis include the fact that the pooled population analysed in this meta-analysis included a limited number of subjects because the sample size in some of the clinical trials was small, and the treatment duration of many of the trials was short. Most of the studies which showed significant rise in TSH and improvement in cardiovascular risk markers were conducted by the group at Hull, UK who has performed this meta-analysis^18–20,36^ but they have done most studies in patients with diseased states such as type 2 diabetes and also in patients with partly compromised thyroid function such as subclinical hypothyroidism at baseline^18–20,36^. There was only a modest 10% increase in TSH which may not be relevant in healthy population but could have a clinically significant effect of patients with compromised thyroid function such as subclinical hypothyroidism. Based on this, further research and analysis is recommended including further investigation involving subclinical hypothyroid patients, not only because of the possible effects on thyroid function but due to potential multiple beneficial effects including cardiovascular health, metabolic syndrome, diabetes, bone health and post-menopausal symptoms^19,45^.

In conclusion, this meta-analysis suggests that soy supplementation has no effect on the thyroid hormones and modestly raises TSH levels, the clinical significance, if any, of the rise in TSH is unclear.

## Supplementary information

Appendix 1,2 & 3

## References

[1] Messina M, Ho S, Alekel DL (2004). Skeletal benefits of soy isoflavones: a review of the clinical trial and epidemiologic data. Curr Opin Clin Nutr Metab Care.

[2] Gil-Izquierdo A (2012). Soy isoflavones and cardiovascular disease epidemiological, clinical and -omics perspectives. Curr Pharm Biotechnol.

[3] Jayagopal V (2002). Beneficial effects of soy phytoestrogen intake in postmenopausal women with type 2 diabetes. Diabetes Care.

[4] Cederroth CR, Nef S (2009). Soy, phytoestrogens and metabolism: A review. Mol Cell Endocrinol.

[5] Kang X, Zhang Q, Wang S, Huang X, Jin S (2010). Effect of soy isoflavones on breast cancer recurrence and death for patients receiving adjuvant endocrine therapy. CMAJ.

[6] Hwang YW, Kim SY, Jee SH, Kim YN, Nam CM (2009). Soy food consumption and risk of prostate cancer: a meta-analysis of observational studies. Nutr Cancer.

[7] Setchell, K. D. Soy isoflavones–benefits and risks from nature’s selective estrogen receptor modulators (SERMs). *J Am Coll Nutr* **20**, 354S-362S; discussion 381S–383S (2001). 10.1080/07315724.2001.10719168 11603644

[8] Nurmi T, Mazur W, Heinonen S, Kokkonen J, Adlercreutz H (2002). Isoflavone content of the soy based supplements. Journal of pharmaceutical and biomedical analysis.

[9] Doerge DR, Sheehan DM (2002). Goitrogenic and estrogenic activity of soy isoflavones. Environ Health Perspect.

[10] Bell DS, Ovalle F (2001). Use of soy protein supplement and resultant need for increased dose of levothyroxine. Endocr Pract.

[11] McCarrison R (1933). A Paper on FOOD AND GOITRE. Br Med J.

[12] Hydovitz JD (1960). Occurrence of goiter in an infant on a soy diet. N Engl J Med.

[13] Chang HC, Doerge DR (2000). Dietary genistein inactivates rat thyroid peroxidase *in vivo* without an apparent hypothyroid effect. Toxicol Appl Pharmacol.

[14] Divi RL, Chang HC, Doerge DR (1997). Anti-thyroid isoflavones from soybean: isolation, characterization, and mechanisms of action. Biochem Pharmacol.

[15] Mazer NA (2004). Interaction of estrogen therapy and thyroid hormone replacement in postmenopausal women. Thyroid.

[16] Messina M, Redmond G (2006). Effects of soy protein and soybean isoflavones on thyroid function in healthy adults and hypothyroid patients: a review of the relevant literature. Thyroid.

[17] EFSA. Risk assessment for peri‐ and post‐menopausal women taking food supplements containing isolated isoflavones. *EFSA journal* (2015).

[18] Sathyapalan T (2017). Soy Reduces Bone Turnover Markers in Women During Early Menopause: A Randomized Controlled Trial. J Bone Miner Res.

[19] Sathyapalan T, Javed Z, Rigby AS, Kilpatrick ES, Atkin SL (2017). Soy Protein Improves Cardiovascular Risk in Subclinical Hypothyroidism: A Randomized Double-Blinded Crossover Study. J Endocr Soc.

[20] Sathyapalan T (2011). The effect of soy phytoestrogen supplementation on thyroid status and cardiovascular risk markers in patients with subclinical hypothyroidism: a randomized, double-blind, crossover study. J Clin Endocrinol Metab.

[21] Sutton, A. J., Abrams, K. R., Jones, D. R., Sheldon, T. A. & Song, F. *Methods for meta-analysis in medical research*., (Chichester: John Wiley & Sons, Ltd., 2000).

[22] Duval S, Tweedie R (2000). Trim and fill: A simple funnel-plot-based method of testing and adjusting for publication bias in meta-analysis. Biometrics.

[23] Messina M, Nagata C, Wu AH (2006). Estimated Asian adult soy protein and isoflavone intakes. Nutr Cancer.

[24] Bruce B, Messina M, Spiller GA (2003). Isoflavone supplements do not affect thyroid function in iodine-replete postmenopausal women. J Med Food.

[25] Dillingham BL, McVeigh BL, Lampe JW, Duncan AM (2007). Soy protein isolates of varied isoflavone content do not influence serum thyroid hormones in healthy young men. Thyroid.

[26] Mittal N (2011). Evaluation of effect of isoflavone on thyroid economy & autoimmunity in oophorectomised women: a randomised, double-blind, placebo-controlled trial. Indian J Med Res.

[27] Bitto A (2010). Genistein aglycone does not affect thyroid function: results from a three-year, randomized, double-blind, placebo-controlled trial. J Clin Endocrinol Metab.

[28] Yim, C. H. *et al*. Effects of isoflavones on thyroid function in premenopausal women. *Thyroid*, S-45-S-136, 10.1089/thy.2007.1519 (2007).

[29] Khaodhiar L (2008). Daidzein-rich isoflavone aglycones are potentially effective in reducing hot flashes in menopausal women. Menopause.

[30] Lazarevic B (2011). Efficacy and safety of short-term genistein intervention in patients with localized prostate cancer prior to radical prostatectomy: a randomized, placebo-controlled, double-blind Phase 2 clinical trial. Nutr Cancer.

[31] Persky VW (2002). Effect of soy protein on endogenous hormones in postmenopausal women. Am J Clin Nutr.

[32] Steinberg FM (2011). Clinical outcomes of a 2-y soy isoflavone supplementation in menopausal women. Am J Clin Nutr.

[33] Tousen Y (2011). Natural S-equol decreases bone resorption in postmenopausal, non-equol-producing Japanese women: a pilot randomized, placebo-controlled trial. Menopause.

[34] Duncan AM (1999). Modest hormonal effects of soy isoflavones in postmenopausal women. J Clin Endocrinol Metab.

[35] Duncan AM (1999). Soy isoflavones exert modest hormonal effects in premenopausal women. J Clin Endocrinol Metab.

[36] Sathyapalan T (2017). Effect of Soy in Men With Type 2 Diabetes Mellitus and Subclinical Hypogonadism: A Randomized Controlled Study. J Clin Endocrinol Metab.

[37] Ryan-Borchers, T. *The Effects Of Soy Isoflavones On Immune And Thyroid Function In Postmenopausal Women* PhD thesis, Washington State University (2004).

[38] Van Wyk JJ, Arnold MB, Wynn J, Pepper F (1959). The effects of a soybean product on thyroid function in humans. Pediatrics.

[39] Divi RL, Doerge DR (1996). Inhibition of thyroid peroxidase by dietary flavonoids. Chem Res Toxicol.

[40] Chang HC, Churchwell MI, Delclos KB, Newbold RR, Doerge DR (2000). Mass spectrometric determination of Genistein tissue distribution in diet-exposed Sprague-Dawley rats. J Nutr.

[41] Wilgrus, H. S. J., Gassner, F. X., Patton, A. R. & Gustavson, R. G. The goitrogenicity of soyabeans. *J Nutr*. **22-43-52** (1941).

[42] Klein RZ (1991). Prevalence of thyroid deficiency in pregnant women. Clin Endocrinol (Oxf).

[43] Canaris GJ, Manowitz NR, Mayor G, Ridgway EC (2000). The Colorado thyroid disease prevalence study. Arch Intern Med.

[44] Haddow JE (1999). Maternal Thyroid Deficiency during Pregnancy and Subsequent Neuropsychological Development of the Child. N Engl J Med.

[45] Messina, M. S and Health Update: Evaluation of the Clinical and Epidemiologic Literature. *Nutrients* **8**, 10.3390/nu8120754 (2016). 10.3390/nu8120754 PMC5188409 27886135

