# Supplementary material for: Systematic Review and Meta-analysis on the Effect of Soy on Thyroid Function
Source: Sci Rep. 2019 Mar 8;9:3964. doi: 10.1038/s41598-019-40647-x (PMC6408586; doi:10.1038/s41598-019-40647-x)
Supplement: Supplementary file 1 — Appendix 1,2 & 3 [file 41598_2019_40647_MOESM1_ESM.pdf]

Systematic Review and Meta-analysis on the Effect of Soy on Thyroid Function – Supplementary Information

Short title: Effect of soy on thyroid

Jemiliat Otun[1], Amirhossein Sahebkar[2,3, 4,], Linda Östlundh[5], Stephen L Atkin[6], Thozhukat Sathyapalan[1]

[1]. Academic Diabetes, Endocrinology and Metabolism, Hull York Medical School, University of Hull
[2.] Neurogenic Inflammation Research Center, Mashhad University of Medical Sciences, Mashhad, Iran
[3.] Biotechnology Research Center, Pharmaceutical Technology Institute, Mashhad University of Medical Sciences, Mashhad, Iran
[4]. School of Pharmacy, Mashhad University of Medical Sciences, Mashhad, Iran
[5.] National Medical Library, College of Medicine and Health Sciences, UAE University, Al Ain, UAE
[6]. Weill Cornell Medicine, Doha, Qatar

Corresponding Author

Jemiliat Otun
Academic Diabetes, Endocrinology and Metabolism,
Hull York Medical School,
University of Hull
Email: hyjo2@hyms.ac.uk

Sponsor: No funding was received for this.

Conflict of interest: No author has any conflict of interest

Appendix 1
Literature Search
Table showing full search log including search string, results and notes.

Academic Databases

Source and date	Search string	Result	Notes
**PubMed**<br><br>**Coverage:**<br><br>1879-<br><br>**Search Date**<br><br>2016-08-01	((TSH[Title/Abstract]) OR ((("receptors, thyrotropin"[Mesh] OR "Iodide peroxidase"[Mesh] OR "TPO protein, human"[Supplementary Concept] OR "thyroid microsomal antibodies"[Supplementary Concept] OR "thyroid dyshormonogenesis 1" [Supplementary Concept] OR "thyroid gland"[Mesh] OR "thyroid hormones"[Mesh] OR "thyroid diseases"[Mesh] OR "receptors, thyroid hormone"[Mesh] OR thyrotropin [Title/Abstract] OR triiodothyronine[Title/Abstract] OR thyroxine[Title/Abstract] OR "iodide peroxidase"[Title/Abstract] OR "TPO protein"[Title/Abstract] OR "TPO proteins"[Title/Abstract]OR thyroid*[Title/Abstract] OR T4[Title/Abstract]) OR T3[Title/Abstract] OR hypothyroid*[Title/Abstract] OR levothyroxine[Title/Abstract])))) AND (((((("genistein combined polysaccharide" [Supplementary Concept] OR "glycitein" [Supplementary Concept] OR "lipofundin s" [Supplementary Concept] OR "soybean oil"[Mesh] OR "soybeans"[Mesh] OR "genistin" [Supplementary Concept] OR "soy foods"[Mesh] OR "sodium-iodide symporter" [Supplementary Concept] OR "daidzein" [Supplementary Concept] OR "phytoestrogens"[Mesh] OR equol[Title/Abstract] OR phytoestrogen*[Title/Abstract] OR genestein[Title/Abstract] OR genistein[Title/Abstract] OR daidzein[Title/Abstract] OR	**1501**	All terms searched in title and abstract and in MeSH when available.<br><br>No Filters or limitations applied<br><br>**Notes for search terms applied:**<br><br>"Hypothyroidism": included under "Thyroid Diseases" in MeSH<br><br>"Thyrotropin" and "Thyrotropin, Beta Subunit" included under: "Receptors, Thyrotropin" in MeSH<br><br>"Thyroid Hormone Receptors alpha" and "Thyroid Hormone Receptors beta" are included under "Receptors, Thyroid Hormone" in MeSH<br><br>"Thyroxine","Triiodothyronine, Reverse" and "Triiodothyronine" are included under: "Thyroid Hormones" in MeSH

	glycitein[Title/Abstract] OR soy*[Title/Abstract] OR genistin[Title/Abstract] OR “lipofundin s”[Title/Abstract] OR "sodium-iodide symporter"[Title/Abstract] OR GCP[Title/Abstract]))) OR "phytoestrogens" [Pharmacological Action])) OR ((isoflavones[Title/Abstract]) OR "Isoflavones"[Mesh]))		"Levothyroxine" refers to “Thyroxine”, which is included under “Thyroid Hormones” in MeSH<br><br>"Genistein" and "Equol" are included under "Isoflavones" in Mesh
**EMBASE**<br><br>(OVID)<br><br>**Coverage:**<br><br>1974-<br><br>**Search Date**<br><br>2016-07-17	((TSH OR thyrotropin OR triiodothyronine OR thyroxine or "Iodide peroxidase") ab. OR (TSH OR thyrotropin OR triiodothyronine OR thyroxine OR "Iodide peroxidase") OR ("TPO protein" OR "TPO proteins").ab. OR ("TPO protein" OR "TPO proteins").ti. OR (thyroid* or T4 OR T3 or hypothyroid* OR levothyroxine).ab. OR (thyroid* or T4 or T3 OR hypothyroid* OR levothyroxine).ti.) OR (thyrotropin receptor/ OR thyrotropin/ OR thyrotropin beta subunit/ OR thyroid peroxidase antibody/ OR iodide peroxidase/ OR thyroid gland/ OR thyroid hormone/ OR thyroxine/ OR 3,3',5' triiodothyronine/ OR liothyronine/ OR levothyroxine/ OR thyroid disease/ OR hypothyroidism/ OR thyroid hormone receptor/) AND (equol OR phytoestrogen*OR genestein or genistein or daidzein or glycitein or soy* or genistin or "lipofundin s" or "sodium-iodide symporter" or GCP or isoflavones).ab. or (equol or phytoestrogen*OR genestein or genistein or daidzein or glycitein or soy* or genistin or "lipofundin s" or "sodium-iodide symporter" or GCP OR isoflavones).ti.OR (equol/ OR glycitein/ OR lipofundin S/ OR soy food/ OR soybean oil/ OR soybean/ OR genistin/ OR soybean milk/ OR soybean protein/ OR sodium iodide symporter/ OR daidzein/ OR phytoestrogen/ OR isoflavone derivative/ OR genistein/ OR thyroid dysgenesis/ OR thyroid hormone receptor alpha/ OR	**1885**	All terms searched in title and abstract (here marked with ti. and ab.) and in MeSH (here marked with /)when available<br><br>No Filters or limitations applied<br><br>**Notes for search terms applied:**<br><br>Supplementary Concepts applied in PubMed: "TPO Protein", "Human, Thyroid Microsomal Antibodies", "Glycitein", "Lipofundin S", "Genistin", "Sodium-iodide Symporter", "Daidzein" and "Pharamlogical Action", "Phytoestrogens" not available in the Emtree<br><br>“Genistein Combined Polysaccharide” is mapped to, and included under “Genistein” in the Emtree<br><br>“Thyroid Dyshormonogenesis” is mapped to, and included under “Thyroid Dysgenesis” in the Emtree<br><br>"Thyroid Peroxidase Antibody" included

	thyroid hormone receptor beta/))		in the Emtree<br><br>"Triiodothyronine, Reverse" mapped to "3,3',5' triiodothyronine" in the Emtree<br><br>“Triiodothyronine” is mapped to “liothyronine” in the Emtree<br><br>Levothyroxine is included in the Emtree<br><br>“Isoflovanes” mapped to “Isoflovane Derivateive” in the Emtree<br><br>“Thyroxine”**,** “Thyrotropin Beta Subunit”, "Thyroid Hormone Receptors Alpha", "Hypothyroidism", "Thyroid Hormone Receptors Beta", “Soy Bean Protein", "Soy Bean Milk", "Genistein", and "Equol" are searched separately in the Emtree<br><br>"Thyroid Dyshormonogenesis 1" is mapped to: "Thyroid Dysgenesis” in the Emtree
**Medline**<br><br>(Web of Science)<br><br>**Coverage:**<br><br>1950-	((TOPIC: (TSH OR thyrotropin OR triiodothyronine OR thyroxine OR "Iodide peroxidase" OR "TPO protein" OR "TPO proteins" OR thyroid* OR T4) OR T3) OR hypothyroid* OR levothyroxine) OR MH= Receptors, Thyrotropin OR Iodide Peroxidase OR Thyroid Gland OR Thyroid Hormones OR Thyroid Diseases OR Receptors, Thyroid Hormone OR Hypothyroidism OR Thyrotropin) AND TOPIC: (equol OR phytoestrogen*OR genestein OR genistein OR daidzein OR glycitein OR soy* OR genistin OR "lipofundin s" OR	**1428**	All terms searched in topic (here marked with TOPIC which includes the fields title and abstract) and in MeSH (here marked with MH) when available<br><br>No Filters or limitations applied<br><br>**Notes for search terms applied:**

**Search Date:**<br><br>2016- 08-01	"sodium-iodide symporter" OR GCP OR isoflavones) OR MH= (Soybean Oil OR Soybeans OR Soy Foods OR Phytoestrogens OR Isoflavones))		Supplementary Concepts applied in PubMed: "TPO Protein, Human", "Thyroid Microsomal Antibodies", "Thyroid Dyshormonogenesis", "Genistein Combined Polysaccharide", "Glycitein", "Lipofundin S", "Genistin", "Sodium-iodide Symporter", "Daidzein" and Pharamlogical Action, "Phytoestrogens not available in MEDLINE's MeSH<br><br>"Levothyroxine" is mapped to "Thyroxine" in MEDLINE's MeSH<br><br>"Hypothyroidism" and "Thyrotropin" are included as separate MEDLINE's MeSH terms<br><br>"Thyrotropin, Beta Subunit" are included under "Thyrotropin"in MEDLINE's MeSH<br><br>"Thyroid Hormone Receptors Alpha" and "Thyroid Hormone Receptors Beta" are included under "Receptors, Thyroid Hormone" in MEDLINE's MeSH<br><br>"Genistein" and "Equol" are included under "Isoflavones" in MEDLINE's MeSH
**Cochrane**	((ti,ab,kw: TSH or thyrotropin or triiodothyronine or thyroxine or "Iodide peroxidase" or "TPO protein" or "TPO proteins" or thyroid* or	**111**	All terms searched in the field for title, abstract and keyword (here marked with

**Coverage:** All databases and all publication years are included<br><br>**Search Date:** 2016- 07-19	T4 or T3 or hypothyroid* or levothyroxine) OR (MeSH descriptor: Receptors, Thyrotropin OR Iodide Peroxidase OR Thyroid Gland OR Thyroid Hormones OR Thyroid Diseases OR Receptors, Thyroid Hormone) AND (ti,ab,kw: equol or phytoestrogen*OR genestein or genistein or daidzein or glycitein or soy* or genistin or "lipofundin s" or "sodium-iodide symporter" or GCP or isoflavones) OR MeSH descriptor: [Soybean Oil] OR [Soybeans] OR [Soybean Proteins] OR [Soy Milk] OR [Phytoestrogens] OR [Isoflavones] OR [Equol] OR [Genistein] OR [Hypothyroidism] OR [Thyrotropin] OR [Thyrotropin, beta Subunit] OR [Thyroid Hormone Receptors alpha] OR [Thyroid Hormone Receptors beta] OR [Thyroxine] OR<br>OR [Triiodothyronine, Reverse] OR [Triiodothyronine]))		ti,ab,kw) and in MeSH (here marked with "MeSH descriptor") when available<br><br>No Filters or limitations applied<br><br>**Notes for search terms applied:**<br><br>Supplementary Concepts applied in PubMed: "TPO Protein, Human", Thyroid Microsomal Antibodies", "Thyroid Dyshormonogenesis", "Genistein Combined Polysaccharide", "Glycitein", "Lipofundin S", "Genistin", "Sodium-iodide Symporter", "Daidzein" and Pharamlogical Action, "Phytoestrogens" not available in Cochrane's MeSH<br><br>"Soy Bean Protein", "Soy Milk" (used for "Soy Bean Milk"), "Equol", "Genistein", "Hypothyroidism", "Thyrotropin", "Thyrotropin, Beta Subunit", "Thyroid Hormone Receptors Alpha", "Thyroid Hormone Receptors Beta", "Thyroxine","Triiodothyronine, Reverse" and "Triiodothyronine" are searched separately in Cochrane's MeSH<br><br>"Levothyroxine" refers to "Thyroxine", which is included under "Thyroid

			Hormones” in Cochrane’s MeSH<br><br>"Thyroxine","Triiodothyronine, Reverse" and "Triiodothyronine" are included under: “Thyroid Hormones” in Cochrane’s MeSH
**Total references retrieved**		**4925**	
**Total references after de-duplication**		**1816**	

Updated search in PubMed, 2018-08-01

Source and date	Search string	Result	Notes
**PubMed**<br><br>**Search Date**<br><br>2018- 08-01	((TSH[Title/Abstract]) OR ((("receptors, thyrotropin"[Mesh] OR "Iodide peroxidase"[Mesh] OR "TPO protein, human"[Supplementary Concept] OR "thyroid microsomal antibodies"[Supplementary Concept] OR "thyroid dyshormonogenesis 1" [Supplementary Concept] OR "thyroid gland"[Mesh] OR "thyroid hormones"[Mesh] OR "thyroid diseases"[Mesh] OR "receptors, thyroid hormone"[Mesh] OR thyrotropin [Title/Abstract] OR triiodothyronine[Title/Abstract] OR thyroxine[Title/Abstract] OR "iodide peroxidase"[Title/Abstract] OR "TPO protein”[Title/Abstract] OR "TPO proteins”[Title/Abstract]OR thyroid*[Title/Abstract] OR T4[Title/Abstract]) OR	**145**	An update of search 1 above.<br><br>Publication filter for 2016- 08-01

	T3[Title/Abstract] OR hypothyroid*[Title/Abstract] OR levothyroxine[Title/Abstract])))) AND (((((("genistein combined polysaccharide" [Supplementary Concept] OR "glycitein" [Supplementary Concept] OR "lipofundin s" [Supplementary Concept] OR "soybean oil"[Mesh] OR "soybeans"[Mesh] OR "genistin" [Supplementary Concept] OR "soy foods"[Mesh] OR "sodium-iodide symporter" [Supplementary Concept] OR "daidzein" [Supplementary Concept] OR "phytoestrogens"[Mesh] OR equol[Title/Abstract] OR phytoestrogen*[Title/Abstract] OR genestein[Title/Abstract] OR genistein[Title/Abstract] OR daidzein[Title/Abstract] OR glycitein[Title/Abstract] OR soy*[Title/Abstract] OR genistin[Title/Abstract] OR “lipofundin s”[Title/Abstract] OR "sodium-iodide symporter"[Title/Abstract] OR GCP[Title/Abstract]))) OR "phytoestrogens" [Pharmacological Action])) OR ((isoflavones[Title/Abstract]) OR "Isoflavones"[Mesh]))		
**Total references after de-duplication**		**145**	

Grey Sources

Date	Source	Search string	Result	Notes
2016- 08- 03	**ProQuest Dissertation and Theses**	TSH OR thyrotropin OR triiodothyronine OR thyroxine OR "Iodide peroxidase" OR "TPO protein” OR "TPO proteins” OR thyroid* OR T4 OR T3 OR hypothyroid* OR levothyroxine AND equol OR phytoestrogen*OR genestein OR genistein OR daidzein OR glycitein OR soy* OR genistin OR “lipofundin s” OR "sodium-iodide symporter" OR GCP OR isoflavones	48	Searched in the field for “Abstract.<br><br>Filters for:<br><br>“Doctoral Dissertation” and “Human Studies” applied.
2016- 08- 03	**Ethos** (Doctorial theses from British Library)	((thyroid AND (soy OR phytoestrogen OR isoflavones OR equol))	0	Searched in the field for “Abstract” and “Theses Title”
2016- 08- 03	**Open Grey** (Grey Literature in Europe)	(thyroid AND (soy OR phytoestrogen OR isoflavones OR equol))	0	Limited search functions available
2016- 08- 03	**Clinical Trials.gov**	((thyroid AND (soy OR phytoestrogen OR isoflavones OR equol))	3	Limited search functions available<br><br>Limitation: “Studies With

				Results" applied
2016- 08- 03	**The New York Academy of Medicine Grey Literature report.**	((thyroid AND (soy OR phytoestrogen OR isoflavones OR equol))	0	Searched in the field for "Title" and "Subject Headings"
**Total references retrieved**			**51**	
**Total references after de-duplication**			**51**	

Appendix 2
Baseline Characteristics

Appendix 2: Data extraction of included studies: Identification, Method and Baseline Characteristics

Identification			Method			Baseline Characteristics									
First Author	Duration	Recruitment Location	RCT Design	Inclusion Criteria Stated	Exclusion Criteria Stated	Sex	Postmenopausal/ Premenopausal	Mean BMI kg/m2			Mean age			Ethnicity	Medical status
								Intervention Group(s)		Pl	Intervention Group(s)		Pl		
**Bitto, A 2010**	3 y	Italy	Parallel	yes	yes	F	Postmenopausal	24.3		25.6	53.8		53.5	Not specified	NSMC
**Bruce, B 2003**	180 d	USA	Parallel	yes	yes	F	Postmenopausal	20.5		20.2	68.9		69.9	W:22%	NSMC
**Dillingham, A 2007**	57 d	Canada	Crossover	yes	yes	M	N/A	25.1	25.3	25.3	27.9			Not specified	NSMC
**Duncan, A 1999**	3 Menstrual cycles	USA	Crossover	yes	yes	F	Premenopausal	22.8	22.8	23.1	63.8	63.9	64.6	Not specified	NSMC
**Duncan, A 1999**	279 d	USA	Crossover	yes	yes	F	Postmenopausal	25.3	25.4	25.4	56.9			Not specified	NSMC
**Jayagopal, V 2002**	12 w	United Kingdom	Crossover	yes	yes	F	Postmenopausal	32.2			62.5			Not specified	Type 2 Diabetic
**Khaodhiar, L 2008**	12 w	USA	Parallel	yes	yes	F	Postmenopausal	29.9	28.3	27.4	52.2	53.2	53.8	W: 62%* AA:30%* O: 8%*	NSMC
**Lazarvic, B 2011**	3-6 w	Norway	Parallel	yes	yes	M	N/A	26.4		25.9	60.7		58.4	Not specified	Localised Prostate Cancer
**Mittal, N 2011**	12 w	India	Parallel	yes	yes	F	Not specified	24.99		24.94	49.14		46.28	IN: 100%Z	Bilateralooph o-rectomy
**Persky, V 2002**	6 m	USA	Parallel	yes	yes	F	Postmenopausal	28.0	26.9	29.6	59.3	61.9	61.0	Not specified	Hypercho-lesterolemic
**Sathyapalan, T 2017**	6 m	United Kingdom	Parallel	yes	yes	F	Postmenopausal	26.3		24.6	52		52	W: 100%	NSMC
**Sathyapalan, T 2011**	16w	United Kingdom	Crossover	yes	yes	52F 8M	Not specified	29.4		29.2	57.2			Not specified	Subclinical hypothyroidis m

Appendix 2 continued

Identification			Method			Baseline Characteristics									
First Author	Duration	Recruitm-ent Location	RCT Design	Inclusion Criteria Stated	Exclusion Criteria Stated	Sex	Postmenopausal/ Premenopausal	Mean Age			Mean BMI kg/m2			Ethnicity	Medical status
								Intervention Group(s)		Pl	Intervention Group(s)		Pl		
**Steinberg, F 2011**	2 y	USA	Parallel	yes	yes	F	Postmenopausal	54.9	54.5	55.0	25.3	24.9	25.4	Not specified	NSMC
**Tousen, Y 2011**	12 m	Japan	Parallel	yes	yes	F	Postmenopausal	53.6*		52	22.0		21.9	JP:100%	NSMC
**Yim, C 2007**	3 m	South Korea	Parallel	yes	no	F	Premenopausal	37.3			Not recorded			K:100%	NSMC
**Sathyapalan, T 2017**	16 w	United Kingdom	Crossover	yes	yes	53F 27M	Not specified	55.1			28.8			Not specified	Subclinical hypothyroidism
**Sathyapalan, T 2017**	3 m	United Kingdom	Parallel	yes	no	M	N/A	52			31.8		31.6	W: 100%	Type 2 Diabetes Mellitus and Subclinical Hypogonadism
**Ryan-Borchers, T 2004**	16 w	USA	Parallel	yes	yes	F	Postmenopausal	56.1	55.9	55.4	27.9	28	28.5	W: 94% A:4% AI: 2%	NSMC

*:Average of total intervention groups, AA: African American, A: Asian, AI: American Indian, B: Black, C: Chinese, IN: Indian, JP: Japanese, NSMC: No significant medical conditions, Pl: Placebo, W: White/Caucasian, WH: White Hispanic, K: Korean, O: Other racial background, Z: Assumption based on the origin of the study, d:days, m: months, y:years

Appendix 3
Intervention and Control/Placebo Characteristics

Appendix 3: Data extraction of included studies: Identification and Intervention

Identification	Intervention						
**First Author**	**Amount given**	**Placebo/Control Type**	**Measurement interval**	**Intervention Group Number (n)**	**Placebo Group Number (n)**	**Supplement/ Whole Soy**	**No intervention groups (including placebo)**
**Bitto, A**	54mg/day	Tablets appeared similar and had a similar taste. The tablets also contained calcium carbonate (500 mg) and vitamin D3 (400 IU)	3 years	40	37	SupplementG	2
**Bruce, B**	50mg 3 times/day	Maltodextrin with 10% caramel colour	90 days	22	16	SupplementI	2
**Dillingham, A**	Low: 1.64±0.19mg/day High: 61.7±7.4mg/day	Milk Protein Isolate	29 days	35 in total		SupplementI	3
**Duncan, A**	Low: 64 ± 9.2mg/day High: 128±16mg/day	0.11 ± 0.01mg isoflavone powder	Days 2–5 of menstrual cycles 3 and 4	14 in total		SupplementI	3
**Duncan, A**	Low: 64 ± 9.2mg/day High: 128±16mg/day	0.11 ± 0.01mg isoflavone powder	93 days	n= 18, except for the low-iso diet period, for which n=17		SupplementI	3
**Jayagopal, V**	30 g of isolated soy protein with 132 mg of isoflavones	30 g of pure microcrystalline cellulose	12 weeks	32 in total		SupplementI	2

* : Total for each thyroid hormone control group, EQ: Equol Supplement, G: Genistein supplement, HI: Hypocotyl isoflavones supplement, I: Isoflavone supplement, IF:Isoflavones, PHY: Phytoestrogen, SP: soy protein powder

Appendix3 continued

Identification	Intervention							
**First Author**	**Amount given**	**Placebo/Control Type**	**Measurement interval**	**Intervention Group Number (n)**		**Placebo/Control Group Number (n)**	**Supplement/ Whole Soy**	**No intervention groups (placebo included)**
**Khaodhiar, L**	Low: 40 mg DRI /day High: 60 mg DRI /day	Soft-gel capsule containing 0g of concentrated isoflavone	4 weeks	Low<br>48	High<br>49	45	Supplement[I]	3
**Lazarvic, B**	30mg/day	Identical in both capsule and container appearance	Not stated	23		17	Supplement[G]	2
**Mittal, N**	75 mg/day	Identical in size, shape and colour and contained identical amounts if the supplementary micronutrients in the intervention tablets	12 weeks	21		22	Supplement[I]	2
**Persky, V**	Low: 56mg/day High: 90mg/day	Casein (0 mg total isoflavones/g protein	3 months	24	22	25,26,24*	Supplement[I]	3
**Sathyapalan, T**	Control: 7.5 g SP, 0mg IF High: 7.5 g SP, 33mg IF Twice a day	7.5 g of the isolated soy protein (0g isoflavone)	6 months	100		100	Supplement[I]	2
**Sathyapalan, T**	Low: 30 g SP, 2mg PHY High: 30g SP, 16mg PHY, Once a day	No control or placebo	3 months	48		48	Supplement[SP]	2

* : Total for each thyroid hormone control group, EQ: Equol Supplement, G: Genistein supplement, HI: Hypocotyl isoflavones supplement, I: Isoflavone supplement, IF:Isoflavones, PHY: Phytoestrogen, SP: soy protein powder

Appendix 3 continued

Identification	Intervention						
**First Author**	**Amount given**	**Placebo/Control Type**	**Measurement interval**	**Intervention Group Number (n)**	**Placebo/Control Group Number (n)**	**Supplement/ Whole Soy**	**No intervention groups (placebo included)**
**Steinberg, F**	Low: 80mg/day<br>High:120mg/day	Placebo tablets: Filler materials and common processing aids.	1 year	135	134	Supplement[HI]	3
**Tousen, Y**	Low: 2mg/day<br>Medium:6mg/day<br>High: 10mg/day	Lactose tablets identical in appearance to intervention tablets.	6 months	Low: 25<br>Medium: 23<br>High: 24	21	Supplement[EQ]	4
**Yim, C**	120mg/day	Not stated	Not stated	34	20	Supplement[I]	2
**Sathyapalan, T**	30 g isolated soy (isoflavone-free) protein/day	30 g casein protein /day	8 weeks	80	80	Supplement[SP]	2
**Sathyapalan, T**	15 g soy protein with 66 mg of isoflavones/ day	15 g soy protein alone without any isoflavones/day	3 months	85	86	Supplement[I]	2
**Ryan-Borchers, T**	Soy Milk: 80 mg isoflavones/day<br>Cow's Milk: 80 mg isoflavones tablet/day.	Cow's milk and placebo supplement	16 weeks	Soy Milk: 25<br>Cow's Milk &Supplement: 25	27	Supplement[I]	3

* : Total for each thyroid hormone control group, A: Implied from the paper (not stated), EQ: Equol Supplement, G: Genistein supplement, HI: Hypocotyl isoflavones supplement, I: Isoflavone supplement, IF:Isoflavones, PHY: Phytoestrogen, SP: soy protein
